# Exploring Benefits for Tibetan Cleft Lip and Palate Recipient Families From the Social Perspective of Healthcare Linkage

**DOI:** 10.1111/hex.70193

**Published:** 2025-02-22

**Authors:** Jing Lin, Xiao Qiong Teng, Chen Xin Zhang, Jing Peng, Jun Yi Guo

**Affiliations:** ^1^ School and Hospital of Stomatology Wenzhou Medical University Wenzhou Zhejiang China; ^2^ The Second Affiliated Hospital of Wenzhou Medical University Zhejiang Province China; ^3^ Wenzhou TCM Hospital of Zhejiang Chinese Medical University Zhejiang Province China; ^4^ Cixi Biomedical Research Institute Wenzhou Medical University Zhejiang Province China; ^5^ Chongqing Mental Health Center Chongqing China

**Keywords:** child with cleft lip and palate, doctor–patient social relationship, medical staff, qualitative research, sense of benefit, Social and medical benefit activities

## Abstract

**Objectives:**

To understand the psychological characteristics and actual sense of benefit of children with cleft lip and palate (CLP) and their caregivers who participated in social welfare medical activities in Tibet, and to promote the humanistic care and quality of medical services provided by healthcare workers in conjunction with social forces.

**Design:**

Qualitative research through interviews and group discussions. Sample: Interviews with 13 participants in the medical activities for Tibet. Measurements: Thematic analysis. This paper adheres to the Consolidated Criteria for Reporting Qualitative Research (COREQ).

**Results:**

The actual benefits perceived by CLP children and their caregivers who participated in the Tibetan social welfare medical activities could be summarized into five themes, Awareness of Aid Activities, Major Difficulties Faced by Affected Children and Families, Perceptions of Medical Assistance, Benefits of Participating in Medical Aid, and Expectations for Future Aid Activities.

**Conclusion:**

Tibetan families with CLP children have benefited from this medical aid program, but there are many problems. It is necessary to increase the cultivation of humanistic and professional qualities of healthcare professionals and to call for and promote more social forces to play the role of healthcare and social teamwork.

**Patient or Public Contribution:**

In our study, we placed a high value on the participation of patients and the public. During the research design phase, we organized a series of focus group discussions, inviting participants to share their experiences and needs, which helped us more precisely define the research questions and objectives. In the data analysis phase, we established a research discussion group consisting of researchers from diverse backgrounds to ensure that our findings aligned with the actual experiences of patients. Additionally, in the process of writing this manuscript, we also invited experienced senior medical and healthcare professionals to review the document, ensuring that the language used was patient‐friendly and that the content was closely aligned with patient concerns.

## 1 Introduction

This study examines the perceived benefits of public healthcare initiatives for families of children with cleft lip and palate (CLP) in Tibet. CLP boasts a 1%–2% incidence rate in Asia and India, including 1.82% in China and higher in remote western regions [1]. Genetics and environmental factors are primary risk factors, with remote areas' unique challenges increasing neonatal risk. High altitude in China's Guangxi Tibetan Autonomous Region may also contribute to incidence [2].

Despite recent improvements in medical service quality, Tibet still lacks adequate facilities for congenital disease care due to weak services, uneven resources, specialist shortages, inadequate infrastructure, and lower quality [3]. This results in a lack of surgical and postoperative support teams, imposing heavy financial burdens and extending treatment periods for Tibetan families [4, 5].

Operation smile, a public welfare medical initiative, collaborates with professionals and government personnel to assist Tibetan families affected by CLP. It provides comprehensive sequential treatments, including surgery, training, speech therapy, rehabilitation, and psychological intervention, and has successfully reduced local medical and economic burdens, decreased birth defect incidence, and improved living conditions.

The sense of benefit refers to positive psychological experiences as situations improve, enhancing quality of life and mitigating negative emotions [6]. The complex treatment process for CLP can negatively impact children's development and family psychology [7, 8]. Assessing whether interventions introduce unknown variables affecting children's and caregivers' development is crucial [9]. From a healthcare linkage perspective, assessing actual benefits and quality of life improvement for participating families is essential. Medical professionals and social stakeholders should focus more on physical and psychological status and the actual benefits of primary program participants—children with CLP and their caregivers [10].

## 2 Background

### 2.1 Medical Care for Families With CLP

In the medical care domain, existing studies have primarily focused on the psychological experiences of CLP children and their caregivers, alongside corresponding healthcare interventions. During the perioperative period, child‐oriented activities and health education are used to enhance children's psychological state and cognitive experiences. Research has also explored the negative emotional impacts on caregivers and proposed nursing guidance and models. Liu et al. examined the relationship between family resilience in CLP children and caregiver factors, providing a basis for psychological support [11]. Other research offers therapeutic support through a multidisciplinary, family‐centered intervention model to meet the evolving therapeutic needs of CLP children as they grow.

This approach is supported by evidence that family‐centered care can improve health outcomes for children with special healthcare needs. Multidisciplinary care teams, including pediatricians, nurses, plastic surgeons, and speech pathologists, are essential for CLP management. Such teams should be comprehensive, collaborative, and family‐centered to ensure the best possible outcomes for CLP children.

### 2.2 Medico‐Social Support for Families With CLP

Sociological research on CLP highlights the psychological stress and support needs of affected children and caregivers, including treatment experiences, social support, cognitive acceptance, emotional strain, and caregiver–child relationships. Comprehensive support is highlighted as crucial for enhancing the quality of life, as demonstrated by Sun et al., Xun et al., Imani et al., and Yusof and Ibrahim [8, 12, 13, 14]. Additionally, Breuning et al. outline critical service needs in Canada [15].

However, there is a gap in research examining the combined impact of nursing, medical care, and societal interventions on public healthcare for families of CLP children in remote ethnic regions. This paper investigates the effects of medical and social interventions on these families, aiming to understand psychological changes and benefits, provide insights for medical practitioners, promote professional and community involvement, enhance understanding of the condition, guide positive emotions, and support physical and psychological development through medical–societal collaboration.

## 3 Methods

### 3.1 Design and Sample

The study adheres to the COREQ guidelines for qualitative research (Supporting Information S1: File 1) [16]. We employed purposive sampling via a typical case strategy, focusing on CLP children and family caregivers, social volunteers, civil affairs personnel, and medical workers involved in Operation Smile's public welfare medical activities in August 2024 in the Oral and Maxillofacial Surgery ward. The sample size was based on the criterion of information saturation [17].

We enrolled school‐age children (6–12 years) with CLP who participated in “Operation Smile” in Tibet, with normal communication skills; no history of mental illness; and no cognitive, visual, or hearing impairments for whom voluntary participation and guardian consent were obtained [18]. Eligible family caregivers participated in or have participated in the Operation Smile public healthcare activities in Tibet; had no history of mental illness, normal cognitive ability, and ability to communicate; and volunteered for the study. Eligible social volunteers were those who participated in or have participated in the Operation Smile public medical activities in Tibet; those with normal communication skills, no previous history of mental illness, and no cognitive dysfunction; and those who volunteered to participate in this study. Finally, eligible medical staff were those who participated in or have participated in the Operation Smile public healthcare program in Tibet; with ≥ 5 years of experience; and who volunteered for the study.

### 3.2 Data Collection and Analysis

**Figure 1:**
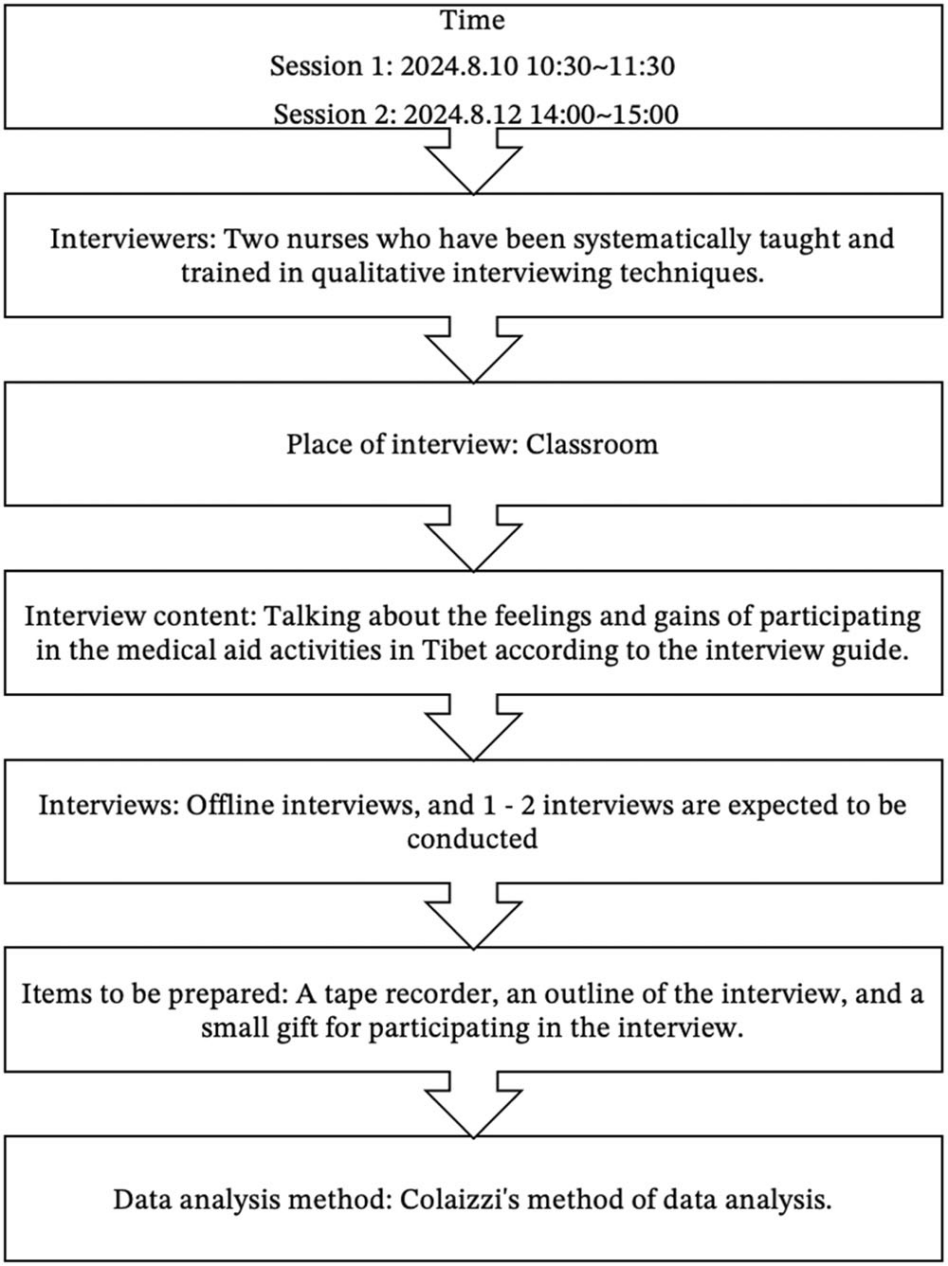
Interview activity roadmap.

According to the research methodology and purpose, the roadmap (Figure 1) and interview guide (Tables 1, 2, 3, 4, 5) for conducting focus group interviews were developed by reviewing domestic and international literature and relevant research groups. A diverse group was selected for the study, including five CLP children, three of their caregivers, a social volunteer, a civil affairs personnel member, and three healthcare workers. The general information of the interviewed children, family caregivers, social workers, and medical staff is shown in Tables 6, 7, 8. Preinterview insights with diverse stakeholders refined our interview guide, with finalization through expert consensus.

**Table 1:** Interview outline for pediatric patients.

Number	Questions
1	Do the adults in your family feel sorry for you or are they afraid to worry about you because of your mouth, and what are their main concerns? Do you worry about that too?
2	Would you be embarrassed to talk to people or would you want to wear a mask because of your mouth and why?
3	Have you ever felt that your life and studies are affected or disturbed by your mouth? Do your classmates and friends make fun of you or bully you?
4	Children who have had surgery: How much do you think you have changed since your previous surgery? What are the main changes?
5	Do you feel special before attending an event like this, and now?
6	How did the adults you came with find out about the availability of this charity event?
7	Have you ever heard of any child participating in this kind of public medical activity before, and what was your reaction at first when you heard about it?
8	What do you think of this charity medical event?
9	What have you found most enjoyable about participating in the program? Or what is the biggest reward?
10	Is there anything more worrisome about being hospitalized for surgery? Do you have confidence in the skills of the doctors and nurses, and are you worried about the results after the surgery?

**Table 2:** Interview outline for caregivers of children with cleft lip and palate.

Number	Questions
1	Did you have any difficulties with food, clothing, and transportation while caring for your child?
2	What attempts and efforts have you and your loved ones (which may include other relatives of the child) made to heal, educate, and take good care of the child? What are the results?
3	How do you and your loved ones (or other relatives) treat your children? How do you communicate? Is there a focus on the child's psycho‐emotional well‐being?
4	What are you and your loved ones thinking about your child's future? What are the concerns and what are the expectations?
5	How do you feel about your child's personality, learning, life skills and interpersonal skills?
6	In caring for your child, what has been the trajectory of your psychological changes from birth to now? Was there ever a sense of powerlessness or frustration? If so, how did you work through and adjust?
7	After attending such an event which do you and your loved ones (including other relatives) think is more important when looking at getting material help or spiritual help and why? If you could choose, what do you think is the most needed support? Why? What do you want more for your child? For example, more material abundance or more moral support?
8	What social support (e.g., government, hospitals, children's agencies, community, etc.) would you most like your child to have in addition to family care? Why?
9	Is there confidence that this act of public service will make a difference and why?
10	How do you feel about the community's efforts to provide this type of public service medical assistance to Tibet?

**Table 3:** Outline of interviews with social volunteers.

Number	Questions
1	What was the initial reason for participating in this charity initiative?
2	What are the main assistance efforts you have been involved in during this activity?
3	What did you get out of such an event? Did you come across any stories that struck a chord with you?
4	What changes do you think this charity has brought to the families of children with cleft lip and palate?
5	Have you ever encountered a situation in which you felt overwhelmed, confused or upset during such an assistance activity?
6	From your point of view, what do you think is going on in the inner world of these children? What do they want from their parents, relatives or the outside world?
7	What are some of the positive forces that you feel when you participate in a charity event like this?
8	As this program continues to grow, where else would you like to see more help for families with children with cleft lip and palate?

**Table 4:** Interview outline for civil affairs department personnel.

Number	Questions
1	In what ways does this program help families with children with cleft lip and palate who are economically disadvantaged?
2	How do you think these pro bono services can help or make a difference to these families?
3	The reason for joining this charity.
4	Have you ever met a child with a cleft lip or palate that struck a chord with you?
5	Have you encountered any difficulties or assistance in this work? Unsupportive, uncooperative, unappreciative, or unenthusiastic assistance from others.
6	Have you encountered any difficulties or assistance in this work? For example, lack of support, lack of cooperation, lack of understanding, or lack of assistance from others?
7	Do you think there are still areas where this charity is not providing enough assistance? Or what areas still need to be strengthened?
8	What are your thoughts on this activity?

**Table 5:** Interview outline for medical personnel.

Number	Questions
1	What was it like to initially come into contact with these children with cleft lip and palate?
2	Has anything happened while caring for or treating them that stands out to you? You can expand on this.
3	Have you gained any positive mental strength from working with these children with cleft lip and palate? You can talk about this in terms of your career, e.g. do you feel that your work and personal values are reflected?
4	In your contact with these children, from your perspective what do you think are the most likely difficulties that these children will encounter as they grow up or in their families? Are there any better solutions or suggestions?
5	What do you think you can do at this pro bono medical event besides helping these children as a medical professional?
6	What do you think are the main ways in which carrying out this charity program will help these special children and how significant is it?
7	Since the launch of this medical charity program, what other aspects do you think need to be improved?

**Table 6:** Basic information of the interviewed child with cleft lip and palate.

Number	Year	Education	Occupation	diagnostic
C1	12	Junior	Student	Palatopharyngeal insufficiency
C2	12	Schoolchild	Student	Postoperative deformity plastic surgery for cleft lip Alveolar synostosis
C3	8	Schoolchild	Student	Alveolar cleft
C4	8	Schoolchild	Student	Alveolar cleft
C5	11	Schoolchild	Student	Postoperative deformity plastic surgery for cleft lip Alveolar synostosis

**Table 7:** Basic information of caregivers for children with cleft lip and palate interviewed.

Number	Year	Relationship with the child	Education	Marital	Occupation	Whether or not sth. is inherited	Diagnostic	Annual family income/$10,000	Medical insurance
Z1	69	Fathers	Primary education	Married	Retired	No	Palatopharyngeal insufficiency	3 ~ 4	Self‐help in the public welfare category
Z2	24	Mother	Tertiary education	Divorce	Clerk	No	Unilateral complete cleft lip Cleft alveolar process Cleft palate Congenital palatopharyngeal insufficiency	3 ~ 4	Self‐help in the public welfare category
Z3	19	Mother	Primary education	Married	Await job placement	No	Unilateral complete cleft lip Cleft alveolar process Cleft palate Congenital palatopharyngeal insufficiency	2 ~ 3	Self‐help in the public welfare category

**Table 8:** Basic information of interviewed medical and social personnel.

Number	Year	Sex	Social roles	Education attainment	Current address	Occupation
N1	29	Female	Civil affairs staff	Undergraduate	Tibet	Officer
N2	14	Female	Volunteers	Junior	Zhejiang	Student
N3	34	Female	Healthcare worker	Undergraduate	Gansu	Nurses
N4	26	Female	Healthcare worker	Bachelor's degree	Zhejiang	Nurses
N5	32	Male	Healthcare worker	Bachelor's degree	Zhejiang	Surgeon

Adoption of a phenomenological research methodology, semi‐structured interviews through focus group interviewing combined with individual interviewing [19, 20, 21]. Given that this study aimed to explore the experiences and perceptions of benefit of Tibetan CLP children and their families, focus group interviews were conducted with five school‐age Tibetan children who participated in Operation Smile, with in‐depth one‐on‐one interviews held with three children and their caregivers. Individual interviews were conducted with three caregivers, and in‐depth one‐on‐one interviews were conducted with the support side, considering the diverse nature of the respondents.

Before interviews, we obtained consent from participants, scheduled interviews, informed participants of the purpose and method, assured confidentiality, and obtained guardian consent for child participants. Interviews took place in a quiet classroom setting within the ward, lasting 30–90 min. The interviews were conducted by two nurses who have been working in clinical oral care for 2 years. Both interviewers had received specialized training in qualitative interviewing techniques.

Audio recordings of the interviews were immediately transcribed into textual materials, which were then sequentially organized and numbered. The interview materials were managed using NVivo 12.0 for analysis, where coding and theme distillation were conducted following Colaizzi's method of data analysis [22].

## 4 Results

Five themes and 13 sub‐themes were distilled (Table 9).

**Table 9:** Main feelings and examples of Tibetan CLP children and their family caregivers participating in Operation Smile.

Overarching theme Themes	Initial themes	Number of mentions	Quotes
Awareness of aid to governance activities	Participants' perceptions of public benefit medical activities in Tibet	21	“It's the first time I've heard of a charity medical event where you can get free surgery.”
	Perceived biases in the plight of CLP children and their families	4	“They all help me, they are nice, everyone is kind, they don't bully me or look down on me because of my mouth, and they are all willing to be friends with me.”
Major difficulties faced by CLP children and their families	Lagging behind in literacy	2	“Still relatively underdeveloped in terms of cultural knowledge.”
	Communication issues	3	“There's still a problem with speech. It's a little slurred, a little nasal.”
	Personality and interpersonal issues	5	“I'm too embarrassed to talk to people, and I even wear a mask to cover my face.”
	Caregiver concerns and emotions	14	“His mom and I were worried that he'd have problems making friends and getting along with his classmates at school.”
	Attempts at resolution	9	“I took him to the local doctor, but it didn't help, so I never got a chance to take him to a big hospital.”
Perceptions of medical services assistance	Perceptions of pro bono medical aid providers	7	“It feels good.”
	Psychological fear of the treatment process	4	“I'm a little afraid of needles.”
	Concerns about the effectiveness of surgical treatment	6	“I'm still confident because the last time the Tibet medical team showed me some photos, the results were still positive. This time, they're coming to treat children with a second repair surgery. The results are also good.”
Gains from participation in medical aid	Improvements at the material and medical levels	10	“It really means a lot to them to be able to change their lives and treat them early when it's the right time to intervene and intervene.”
	Guided by the power of the inner spiritual dimension	13	“I'm lucky to have great support, encouragement, and companionship from those around me, which helps me keep going. It's also important to me that children grow up in a loving environment!”
Expectations for aid to governance activities	Families living in poverty with CLP—intensifying advocacy efforts	3	“It seems that some places are not aware of this program. The last time I went to another village, I met two kids, both just two years old, who hadn't yet had their cleft lip and palate repaired. They said they didn't know about this program.”

### 4.1 Awareness of Aid to Governance Activities

#### 4.1.1 Participants' Perceptions of Public Benefit Medical Activities in Tibet

Most recipients were made aware of the avenues through which families of CLP children in Tibetan regions can access assistance from the government's liaison with medical contractors. Most recipients demonstrated a high level of receptivity to the proposed assistance, exhibiting no discernible resistance to the involvement of medical personnel and the community in this supportive endeavor. A mother of a CLP child said:

I happened to hear a village official talking about a free medical clinic that's been set up by other places to treat children with cleft lips and palates.

Two CLP children were interviewed directly:

This was the first time I heard about the free surgery in a charity medical program, and I was very happy to hear that I could finally have the surgery.

Upon initial contact with CLP children and their families in Tibet, supporters recognized the urgent need for medical and living assistance. Initially repelled by the underdeveloped conditions, they developed a strong desire to provide immediate aid, joining the medical aid operation. They provided financial assistance, facilitated patient transportation to remote medical facilities, employed intermediaries for communication, and offered professional services to assist these families.

I was pretty intolerant; these families with children who have cleft lips and palates are facing economic challenges, transportation difficulties in remote areas, and the fact that the children's caregivers have limited access to education and may not have the necessary skills to bring their children to the clinic for treatment.

#### 4.1.2 Perceived Biases in the Plight of CLP Children and Their Families

Some of the supportive interviewees initially held the stereotypical impression that CLP children in Tibet would have psychological problems due to their physical defects that affect their interpersonal and daily lives and increase the psychological burden on their family caregivers. However, in reality, most CLP children have a positive outlook, and their caregivers are not overly concerned. One of the medics explained:

I initially thought these kids would be particularly self‐conscious and afraid of people, but they're actually very friendly and happy. They don't seem to be bothered by the deformity of their mouths(…). It's not something that's widely understood among the parents because there are quite a few cases of this [cleft lip and palate] in their community. Typically, a family has seven or eight children, one or two of whom have this condition. When you add up the villages and districts, it's a significant number. The parents of the cleft lip and palate children in the Tibet area are also used to it.

### 4.2 Major Difficulties Faced by CLP Children and Their Families

#### 4.2.1 Lagging Behind in Literacy

The advancement of cultural standards not only enhances the inner spiritual realm but also facilitates tangible shifts in economic and medical domains. In Tibet, the economic status of families with CLP children is typically low. Additionally, the cultural level of family caregivers is often limited, and the modern, information‐based medical care model and long‐distance transportation options present significant challenges for these families. One of the participants stated:

Unfortunately, I don't have much cultural knowledge either. I can only read up to elementary school level. I can't even write [my] own Chinese name. This time, we're following the leader's example and coming along too. Some of us can't even speak Mandarin well.

#### 4.2.2 Communication Issues

Two recipient interviewees indicated that, due to congenital physical defects resulting in significant communication challenges, they were unable to vocalize with clarity and had to rely on others to convey information and engage in passive communication for an extended period of time. As one explained:

(…) This was the first surgery, and there are still problems with speech; it can be a little unclear and a little nasal, because it's the inside of the mouth that's split (…) He was also feeling pretty scared and didn't want to talk to anyone, so his mom and sister spent a lot of time chatting with him.

#### 4.2.3 Personality and Interpersonal Issues

In addition to the functional impairment of the maxillofacial region, the congenital deformities of the cleft lip and/or palate in CLP children also give rise to concerns among the children and their families regarding facial esthetics. These concerns may even affect the children's mental health, triggering the development of personality dysfunction, social disorders, and other problems. A child interviewee with CLP stated:

I'm a bit self‐conscious about talking to people because of my mouth. Sometimes, I even wear a mask to cover my mouth.

Some CLP children are constrained in their ability to form friendships due to the physical deformities associated with the condition. Children interviewed mentioned:

(…) I was afraid people would laugh at me, so I played with my classmates at school, but I didn't have many friends who played well with me because I couldn't speak clearly.

#### 4.2.4 Caregiver Concerns and Emotions

The caregivers of CLP children are confronted with a multitude of challenges and stressors in their role of caring for their children. Nutritional, psychological, and medical health issues may arise, and caregivers may exhibit avoidance or coddling behaviors, as well as negative emotions such as worry, guilt, and self‐blame while caring for their children. A few interviewed caregivers of CLP children in Tibetan areas indicated that they would pay particular attention to the issues arising from their child's physical and facial defects during the child's growth process. Additionally, they noted that the development of emotions such as guilt and worry was also related to the development of affection for the child. As one of the caregivers explained:

(…) I'm still concerned that he might have difficulty speaking clearly, and I'm a bit worried about him. He didn't speak much at school since he was a child. When he did speak, it was just ‘ow, ow, ow’—not clear. I would think about what to do and how to stop this, fearing that people would laugh at him and that he wouldn't be able to communicate with other people.

Some caregivers may indicate that the longer they interact with the child, the stronger their relationship appears to become, and the more profound feelings of self‐blame become. A mother of a child said:

As time goes by, I find myself developing stronger feelings for him, and I also feel more guilty. I'll think about how I didn't pay much attention to him when I was pregnant. I didn't go to the ultrasound or other tests, and I was taking medication. When he was born, he was very well‐behaved; I felt powerless and frustrated.

#### 4.2.5 Attempts at Resolution

Before enrolling in this medical charity program, the interviewees had attempted to secure medical care independently. However, financial constraints and a lack of adequate medical resources in their local area compelled them to postpone their plans to seek medical treatment. With regard to the psychological issues that may emerge during a child's development in the context of their physical deformity, caregivers frequently serve as a conduit between their child and the external environment, facilitating positive emotional experiences through parent–child bonding and the influence of external human interactions. As caregiver one explained:

I've taken him to the doctor before because of the mouth problem, but the medical care in my hometown is not very good, so I haven't had the chance to do the surgery until now.

Despite the efforts of caregivers of CLP children to enhance the quality of life for these children and mitigate the adverse effects of the condition, the prevailing healthcare environment, which is characterized by inadequate resources and suboptimal care, continues to impede the timely delivery of interventions for CLP children. As one explained:

His mom was also concerned about him from the time she discovered he had this issue [cleft palate] and took him to the local doctor, who wasn't able to provide a solution. Unfortunately, she never had the opportunity to take him to a larger medical facility after that.

### 4.3 Perceptions of Medical Services Assistance

#### 4.3.1 Perceptions of Pro Bono Medical Aid Providers

Given that the primary participants in medical charity activities are school‐age children, it is imperative to recognize the sensitivity and vulnerability inherent to this unique demographic when interacting with medical and social personnel beyond their immediate families and familiar upbringing. CLP children tend to exhibit reticence and discomfort in their initial interactions with healthcare professionals. However, they demonstrate unanimous acceptance of the altered treatment environment and the healthcare and social professionals who assist them. As the sequence of CLP treatments progresses, a multitude of positive changes emerge in the patient's role and the caregiver's mindset. A participating CLP child said:

Those aunts and uncles looked nice and friendly [grinning, shy].

As treatment progresses, there is an increase in the frequency and depth of communication between the recipient and the provider. This leads to a transformation in the doctor–patient relationship from a purely transactional and passive role to a more complex and collaborative dynamic one. As one nurse said:

Last time, there was a kid in his 20 s who had multiple surgeries for cleft lip and palate, Now, his lip looks so well repaired that he was so grateful to the doctor that he traveled more than 20 h by car, carrying a basket of flowers, and took the train from a faraway place to thank the doctor, and even kneeled down in front of him (…) will feel like they're not just patients, but friends, they'll feel really close to us and feel better in their hearts.

#### 4.3.2 Psychological Fear of the Treatment Process

While infants, toddlers, and children in their formative years typically exhibit a fear of medical treatment, some CLP children who participated in medical activities in Tibet displayed a notable motivation to undergo surgical treatments, despite experiencing disturbances in their self‐image and maladjustment following the procedure. As one child said:

Everything is fine. I'm happy to come here and have the surgery for free. (…) I'm a little afraid of needles (embarrassed laugh).

One nurse said:

Some people may have trouble accepting or adapting to the appearance of their own lips after surgery. After all, at first, the lips are cracked and then they're stitched up. The change is significant, and it can take a little time to get used to them. They might be a little self‐conscious and also worried about what other people will think when they see their stitches, wounds, and so on. They'll probably wear a mask to cover their mouths.

Additionally, the medical personnel who participated in the study observed that CLP infants and children exhibit certain cumulative effects of medical fear and behavioral responses to treatment. A participating healthcare professional explained this phenomenon of the cumulative effect of medical fear in this way:

(…) Young children often have to come to the hospital to have blood drawn by a nurse; they may also be afraid of it. Additionally, since the procedure is usually done multiple times, the first time when they are a few months old, and then again when they are older (…) Even when I'm tending to other patients, I've noticed that babies and toddlers with cleft lips and palates tend to react negatively when they see people wearing our clothes. They often turn their heads to hide in their mothers' arms or just want to cry. It's clear that they're already feeling scared of us.

#### 4.3.3 Concerns About the Effectiveness of Surgical Treatment

Most recipient respondents were not overly concerned about the outcome of the surgical treatment. Caregivers of CLP children were more positive in their expectations of the treatment based on positive social media campaigns and trust in the provider, and CLP children were accepting of the impending cosmetic changes. One mother said:

I'm still confident because the last time the Tibet medical team showed me some photos, the results were still positive. This time, they're coming to treat children with a second repair surgery. The results are also good.

CLP children said:

I'm pleased to say that my speech is much clearer now than it was before I had the surgery. In fact, I'm much better at it now than I was before the operation. The doctor said that it will get better in the future (…) No worries; I have complete confidence in the doctor! I have complete confidence in the doctor's abilities.

### 4.4 Gains From Participation in Medical Aid

#### 4.4.1 Improvements at the Material and Medical Levels

The economic pressure and underdeveloped medical conditions faced by families with CLP children are significant factors influencing their decision to participate in medical aid activities in Tibet. These families often lack the financial resources to bear the burden of transporting their children to cities with superior medical facilities for prolonged periods of treatment. The provision of medical aid enables the resolution of critical medical needs, alleviates economic pressures associated with medical treatment, and enhances the material well‐being of impoverished Tibetan families with CLP children. As one explained:

(…) this program can help them to have the operation done, which can bring them substantial medical help (…) After all, being able to provide treatment to these children free of charge relieves a great financial burden on these families living in poverty.

#### 4.4.2 Guided by the Power of the Inner Spiritual Dimension

The participants in the activity, including supporters and recipients, asserted that the Operation Smile public welfare medical aid activity fostered a sense of unity among individuals from diverse backgrounds. Through the establishment of human connections, the activity enabled the identification and resolution of the needs of Tibetan CLP children and their families. The beneficiaries of this activity were not solely the recipients; both parties emerged from the experience with a renewed sense of strength. As one of them said:

I think it's important to have spiritual support when you're raising a child on your own. Having people around you who can offer encouragement and companionship is also key. And, of course, it's vital to provide a loving environment for your child to grow up in (…) I feel very shocked (…), and the people around me are also very kind.

During the interviews, it was additionally observed that some healthcare professionals were able to augment their sense of professional benefit by engaging in social and medical activities aimed at the treatment of CLP children. One medical staff member said:

From my professional perspective (…), I'll feel like my work is meaningful and that I've made a real difference for them and their families. For example, from a nursing perspective, some parents (…) are unsure about breastfeeding, feeding their child, and the dos and don'ts of infant care. We can provide guidance on these topics, which helps them feel more confident and prepared for their child's discharge from the hospital.

### 4.5 Expectations for Aid to Governance Activities

#### 4.5.1 Families Living Poverty With CLP—Intensifying Advocacy Efforts

The majority of CLP children and their caregivers expressed satisfaction with this social medical activity. However, one caregiver of a CLP child noted that remote areas are relatively inaccessible, the local population is less educated, and obtaining information is challenging. Consequently, there is a need to increase the publicity of Operation Smile's medical activities in Tibet to assist a larger number of individuals in need. This program participant mentioned:

I think we can do a better job of publicizing this [Tibet Aid Medical Activity] so that more people know about it. Some places are still unaware of this activity (…) It seems that some places are not aware of this program. The last time I went to another village, I met two kids, both just 2 years old, who hadn't yet had their cleft lip and palate repaired. They said they didn't know about this program.

## 5 Discussion

The Smile Alliance Medical Social program aims to improve outcomes for children with cleft lip and palate (CLP) through surgical interventions, overcoming challenges posed by geographical and technological barriers. Prenatally diagnosed cases should follow a staged treatment plan, starting with early orthodontic measures and initial surgery within the neonatal period. In Tibetan regions, despite guidelines recommending surgery for CLP children between 2 and 6 months after birth and completion of palate repair by 12 months, most children receive interventions much later, often during childhood, adolescence, or even adulthood. This delay is due to traditional beliefs and limited resources, which result in missed optimal treatment times and compromised repair outcomes. Integration of Western and Tibetan medicine is ongoing, yet a shortage of specialists persists. A comprehensive approach, encompassing surgery, nursing, speech therapy, and psychological support, is essential, underpinned by public welfare initiatives and a collaborative healthcare model to ensure timely and holistic care.

### 5.1 Pay Attention to the Real Emotions and Needs of Children and Their Caregivers and Guide Them to the Right Concepts

Research consistently highlights the significant emotional and psychological burdens faced by CLP children and their caregivers. Studies by Liu et al. (2024), Lentge et al., and Ueki et al. document elevated distress levels among these populations [23, 24, 25]. In contrast, our investigation into Tibetan CLP children found minimal psychological or relational impediments, with caregivers generally displaying affirmative attitudes. This divergence may be attributed to regional characteristics, cultural sophistication, and ethnic nuances, as evidenced by Czajeczny et al.'s work. [26]

Caregivers with lower educational attainment may neglect foundational aspects of child development, while more educated caregivers may experience heightened concerns and anxieties. The condition itself may foster feelings of self‐blame and indulgence, potentially leading to underlying psychological issues, as noted by Cocquyt et al. and Heppner et al. [27, 28]

It is imperative for medical and social volunteers to attune themselves to the unique needs and sentiments of CLP children and their caregivers. Tailored support should address specific familial contexts, facilitate disease management, aid in emotion regulation, and impart efficacious parenting techniques. This approach is supported by evidence that family‐centered care can improve health outcomes for children with special healthcare needs. Multidisciplinary care teams, including pediatricians, nurses, plastic surgeons, and speech pathologists, are essential for CLP management. Such teams are recommended to be comprehensive, collaborative, and family‐centered to ensure the best possible outcomes for CLP children.

### 5.2 Focusing on the Cultivation of Medical Personnel's Humanistic Professionalism

The study found that Tibetan CLP children experience postoperative self‐image issues and medical fear [29, 30]. Moreover, there is a noticeable bias in how medical staff, social volunteers, and civil affairs personnel perceive these children. These groups often interact with children using stereotypes, holding preconceived notions about their self‐esteem, social skills, and caregivers' emotional resilience. Such perceptions can negatively affect medical staff's attitudes and behaviors, leading to decreased professional competence and unmet medical needs [31, 32, 33]. Preconceived notions can widen the doctor–patient status gap, creating psychological barriers that hinder communication, affect patient care experiences, and potentially cause doctor–patient conflicts [34]. Therefore, medical staff should enhance professionalism, avoid subjective judgments, and use both perceptual and rational thinking to assess patients' and caregivers' emotions, providing appropriate humanistic care. Hospitals should also focus on cultivating humanistic professionalism among medical staff.

### 5.3 Increasing the Participation of Medical Staff in Social Welfare Medical Activities and Channeling Positive Forces

In this study, most respondents reported benefiting from the public welfare medical activity supporting Tibet, especially spiritually. Medical personnel effectively meet the specific treatment and care needs of CLP children and their caregivers through medical assistance. Observing community and medical professional collaboration in aiding impoverished Tibetan families with CLP encourages reflection on the individual–society relationship, enhancing professional growth and self‐worth realization. Furthermore, witnessing social and medical professional collaboration in assisting these families promotes reflection on the individual–community interconnection, significantly boosting motivation and contributing to professional advancement and self‐perception. Hospitals are encouraged to actively engage in relevant social welfare medical activities, promoting staff participation at material or spiritual levels. This approach facilitates experience and enthusiasm accumulation among medical staff while transmitting positive social energy.

### 5.4 Advancing the Collaborative Role of Medical and Nursing Societies, Addressing Growing Social Concerns, and Enhancing Medical Care and System Construction in Tibet Are of Paramount Importance

Families of CLP children have significantly contributed to the advancement of professional talent involvement, medical technology, and the optimization of medical services in Tibet. They have also played a crucial role in enhancing health education and preventive healthcare among the local population by disseminating health knowledge and promoting disease prevention and control. The caregiving concepts of these families have been positively guided, and it is recommended that social media platforms increase attention to such public welfare medical activities, expanding publicity and information reach to break geographical barriers. This would allow more families in similar situations to participate and benefit. With the support of medical personnel and community care, these social welfare medical aid activities have facilitated genuine medical treatment and assistance, promoting harmony in doctor–patient and social relations. Consequently, the social government is obliged to initiate initiatives in education, transportation, economy, and medical care to establish a robust foundation. Furthermore, the government must enhance oversight of the quality of each component in the support system and refine relevant mechanisms to ensure that the benefits of social welfare medical care reach all patients and families in genuine need.

## 6 Conclusions

This study employed qualitative research methods, conducting interviews with CLP children, their caregivers, medical staff, and community members involved in Tibet Aid for Social Welfare medical activities. The investigation identified and refined five key themes: Awareness of Aid Activities, Major Difficulties Faced by Affected Children and Families, Perceptions of Medical Assistance, Benefits of Participating in Medical Aid, and Expectations for Future Aid Activities.

The social welfare medical activities in Tibet increase perceived benefits for CLP and their caregivers. Collaboration between community care providers and medical personnel reduces economic and medical burdens, fostering harmonious relationships. However, regional, cultural, and ethnic differences, along with cognitive biases, affect medical service delivery. Recognizing genuine emotions and needs, facilitating positive conceptualizations, and enhancing humanistic and professional capabilities of medical personnel are crucial. The healthcare system in Tibet must improve treatment cycle management and postoperative support, including comprehensive follow‐up and care models, to enhance patient outcomes and system effectiveness. The study is limited by its single‐center nature and small sample size. Future research should expand personnel categories and include multicenter studies.

## Author Contributions

**Jing Lin:** writing – original draft, investigation, methodology, writing – review and editing. **Xiao Qiong Teng:** validation, project administration, writing – review and editing, writing – original draft. **Chen Xin Zhang:** investigation, software, writing – original draft, supervision. **Jing Peng:** investigation, writing – review and editing, project administration, validation, data curation, supervision, software. **Jun Yi Guo:** writing – review and editing, funding acquisition, conceptualization.

## Ethics Statement

This study was reviewed and approved by the Ethics Committee of the School and Hospital of Stomatology, Wenzhou Medical University (Ethics Approval Number: WYKQ2023038). All participants provided written informed consent before their participation in the study. The study was conducted in accordance with the ethical standards laid down in the 1964 Declaration of Helsinki and its later amendments.

## Conflicts of Interest

The authors declare no conflicts of interest.

## Supporting information

Supporting information.

## Data Availability

The data that support the findings of this study are available on request from the corresponding author. The data are not publicly available due to privacy or ethical restrictions.
