# Supplementary material for: Exploring Benefits for Tibetan Cleft Lip and Palate Recipient Families From the Social Perspective of Healthcare Linkage
Source: Health Expect. 2025 Feb 22;28(1):e70193. doi: 10.1111/hex.70193 (PMC11846023; doi:10.1111/hex.70193)
Supplement: Supplementary file 1 — Supporting information. [file HEX-28-e70193-s001.docx]

| **Table I** Consolidated criteria for reporting qualitative studies (COREQ):32-item checklist | |
| --- | --- |
| \| No Item Guide questions/description \| \| --- \| | |
| **Domain 1:Research team and reflexivity** | |
| Personal Characteristics | |
| 1.Interviewer/facilitator | Which author/s conducted the interview or focus group? **This article’authors: Jing Lin and Chen Xin Zhang**  What were the researcher's credentials?E.g.PbD,MD. **Master of Nursing.**  What was their occupation at the time of the study? **Nursing.**  Was the researcher male or female? **Female.** |
| 2.Credentials |  |
| 3.Occupation |  |
| 4.Gender |  |
| 5.Experience and training | What experience or training did the researcher have?  **Researchers have been systematically taught and trained in qualitative techniques.** |
| Relationship with participants | |
| 6.Relationship established | Was a relationship established prior to study commencement?  **Yes.** |
| 7.Participant knowledge of the interviewer | What did the participants know about the researcher?e.g.personalgoals,reasons for doing the researcb.  **Before participating in this study, the participants' consent had been obtained. Relevant matters had been informed, and the informed consent of the participants had been obtained. Therefore, they were aware of the interviewers' occupations and the purpose of conducting this study, as well as their working roles in this study.** |
| 8.Interviewer characteristics | What characteristics were reported about the interviewer/facilitator?e.g.Bias,assumptions, *reasons and interests in the rsearch topic.* ***“children with cleft lip and palate and their families in remote areas with regional characteristics”*** |
| **Domain 2:study design** | |
| Theoretical framework | |
| 9.Methodological and orientation Theory | What methodological orientation was stated to underpin the study?eg.grounded theory, *discourse anabsis,etbnograpby,phenomenology,content anabsis.* **Phenomenological research methods.** |
| Participant selection | |
| 10.Sampling | How were participants selected?eg.purposive,convenience,consecutive,snowball. **“Purposive sampling was conducted through the typical case sampling strategy”** |
| 11.Method of approach | How were participants approached?e.g.face-to-face,telepbone,mail,email. **face-to-face** |
| 12.Sample size | How many participants were in the study?**15** |
| 13.Non-participation  Setting | How many people refused to participate or dropped out?Reasons?**No one.** |
| 14.Setting of data collection | Where was the data collected?e.g.bome,clinic,workplace. **An illustrative classroom in a ward.** |
| 15.Presence of non-participants | Was anyone else present besides the participants and researchers? **No.** |
| 16.Description of sample Data collection | What are the important characteristics of the sample?e.g.demograpbic data,date. **“Two children with cleft lip and palate and their caregivers, one social volunteer and civil affairs personnel, and two healthcare workers were selected to participate in the activities to conduct pre-interviews”** |
| 17.Interview guide | Were questions,prompts,guides provided by the authors?Was it pilot tested? **Yes.** |
| 18.Repeat interviews | Were repeat interviews carried out?If yes,how many? **Yes,two.** |
| 19.Audio/visual recording | Did the research use audio or visual recording to collect the data? **Audio record.** |
| 20.Field notes | Were field notes made during and/or after the interview or focus group? |
| 21.Duration | What was the duration of the interviews or focus group?**“The interviews lasted between 30 and 90 minutes”** |
| 22.Data saturation | Was data saturation discussed? **“Yes,** **The sample size of the study was based on the criterion of information saturation”** |
| 23.Transcripts returned | Were transcripts returned to participants for comment and/or correction? **Yes.** |
| **Domain 3:analysis and findingsz** | |
| Data analysis | |
| 24.Number of data coders | How many data coders coded the data? **18.** |
| 25.Description of the coding tree | Did authors provide a description of the coding tree? **Yes.** |
| 26.Derivation of  themes | Were themes identified in advance or derived from the data? **Derived from the data.** |
| 27.Software | What software,if applicable,was used to manage the data?  **“Nvivo 12.0 software for analysis and coding and distilling themes using Colaizzi's method of data analysis”** |
| 28.Participant checking  Reporting | Did participants provide feedback on the findings? **Yes.** |
| 29.Quotations presented | Were participant quotations presented to illustrate the themes /findings?Was each *quotation identified?e.g.participant number.* **Yes.** |
| 30.Data and findings consistent | Was there consistency between the data presented and the findings? **Yes.** |
| 31.Clarity of major themes | Were major themes clearly presented in the findings? **Yes.** |
| 32.Clarity of minor themes | Is there a description of diverse cases or discussion of minor themes? **Yes.** |
